# The Effects of Trait Anxiety and Emotional Word Type on the Processing of Chinese Words: An ERP Study

**DOI:** 10.3390/bs16010096

**Published:** 2026-01-11

**Authors:** Jia Liu, Lin Fan

**Affiliations:** 1 School of Foreign Studies, Hebei Normal University, Shijiazhuang 050024, China; jiayou0826@hebtu.edu.cn; 2 National Research Center for Foreign Language Education, Beijing Foreign Studies University, Beijing 100089, China; 3 Artificial Intelligence and Human Languages Lab, Beijing Foreign Studies University, Beijing 100089, China

**Keywords:** trait anxiety, emotion-label words, emotion-laden words, ERP

## Abstract

The dissociation between emotion-label and emotion-laden words has been investigated in both behavioral and electrophysiological studies. However, how individual differences modulates the processing of emotional words has not been fully explored. Trait anxiety, as an important individual difference variable, plays a vital role in emotion processing, and may influence the processing of emotional words. To reveal the effects of trait anxiety and emotional word type on the processing of Chinese words, the present study adopted a lexical decision task (LDT) and event-related potential (ERP) technique to collect the behavioral and electrophysiological data from high-trait-anxious (HTA), medium-trait-anxious (MTA) and low-trait-anxious (LTA) individuals. Behaviorally, participants demonstrated longer reaction times (RTs) and lower accuracy (ACC) when processing emotion-laden words, as opposed to emotion-label words and neutral words. Electrophysiologically, both emotion-label and emotion-laden words induced enhanced N170 amplitudes relative to neutral ones. Compared with neutral words, emotion-laden words elicited larger early posterior negativity (EPN) amplitudes in the right hemisphere and increased late positive component (LPC) amplitudes, whereas emotion-label words elicited a stronger N400. EPN amplitudes were modulated by the interaction between trait anxiety and emotional word type. In HTA individuals, emotion-laden words evoked a larger EPN than emotion-label and neutral words, supporting the mediated emotion concept account, density hypothesis, and embodiment emotion account. During the late elaborative processing stage, LTA participants exhibited larger LPC amplitudes than HTA individuals, which aligns with the “vigilance-avoidance” pattern.

## 1. Introduction

Anxiety is defined as a bias toward attending to threat-related stimuli (Edwards et al., 2010). This bias is not exclusive to patients with clinical anxiety diagnoses—it also manifests in healthy individuals with high trait anxiety, who often experience elevated levels of state anxiety (Edwards et al., 2010). According to Spielberger et al. (1983), trait anxiety denotes relatively stable individual differences in anxiety proneness—specifically, differences in people’s tendency to perceive stressful situations as dangerous or threatening, and to respond to these situations with increased intensity of their state anxiety reactions; State anxiety refers to a tangible reaction or ongoing process that occurs at a specific moment and with a particular level of intensity. Trait anxiety influences the process of negative stimuli (Armitage & Eerola, 2025). Existing studies have demonstrated that high-trait-anxious (HTA) participants had an attentional bias for threatening information, which may contribute to the mediation of emotional vulnerability via regulating emotional reactivity to real life events of pressure (Macleod & Hagan, 1992). Hence, the mechanisms underlying emotional word processing in HTA individuals hold considerable potential for informing strategies to improve emotional well-being.

Compared to low-trait-anxious (LTA) individuals, healthy HTA individuals allocated more attentional resources to threatening words (MacLeod & Rutherford, 1992; Rutherford et al., 2004). For example, Miller and Patrick (2000) used startle reflex and reaction time (RT) measures to investigate how HTA and LTA participants processed threatening stimuli. Participants were instructed to name the color of the threatening, pleasant, and neutral words under circumstances of expectation, or no expectation, of electric shock. Between word presentation and naming, there were acoustic startle probes. Results showed that the HTA group responded faster to pleasant than threatening words regardless of the existence of electric shock. Together with the results of startle reflex, the findings could be concluded as that compared to LTA individuals, HTA participants exhibited stronger defensive emotional reactivity to threatening words under the conditions of shock. In addition, HTA participants showed an attentional bias towards threatening words. Edwards et al. (2006) took valence (threatening, nonthreatening words), specificity (electric related, nonelectric related), exposure mode (masked, unmasked), and shock condition (with, without threat of shock) as within-subjects variables and trait anxiety (HTA, LTA groups) as the between-subjects variable to investigate the attentional bias for threatening words of individuals with different levels of trait anxiety. In unmasked trials, HTA individuals exhibited significant color-naming interference for all threatening words under shock threat, but not in the shock-safe circumstance. In masked trials, despite chance performance in identifying lexical status, the HTA group demonstrated facilitated color naming for all threatening words under shock threat, but with no such effect in the shock-safe condition. No similar effects were found in the LTA individuals. Similarly, in an emotional Stroop task (EST), Edwards et al. (2010) found that when unmasked trials preceded masked ones, the HTA group showed a greater attentional bias toward threatening words than the LTA group, which was not modulated by state anxiety. In recent years, Y. Zhao et al. (2023) employed a dot-probe task to examine the attentional bias of anxious individuals. They found that HTA individuals showed an attentional bias toward COVID-19-related words and attentional bias away from general threatening words; LTA individuals exhibited attentional bias away from both types of words.

In terms of emotional word processing, the vast majority of studies classified emotional words according to valence, arousal or the basic emotions. However, Pavlenko (2008) divided emotional words into emotion/emotion-label and emotion-laden words based on emotional prototypicality (EmoPro). She defined emotion-label words as words that directly express or describe emotions such as *happy* and *sad* while emotion-laden words did not express or describe emotions directly, but elicited certain emotion(s) through the connotations of the words, such as *smile* and *punishment*. Emotion-label words’ affective meanings are the same as their conceptual meanings; hence, the affective meaning of emotion-label words could be accessed in a direct way. Emotion-laden words’ affective meanings are different from their conceptual meanings, so the access of their affective meanings might be mediated by the conceptual meanings (Altarriba & Basnight-Brown, 2010). Moreover, emotion-laden words exhibited higher semantic complexity than emotion-label words (Liu et al., 2023).

Most behavioral studies have evidenced the processing differences of these two types of emotional words in various tasks (Altarriba & Basnight-Brown, 2010; Knickerbocker et al., 2015) except two studies including Martin and Altarriba (2017) and Vinson et al. (2014). The inconsistency between behavioral studies might be due to the details of experimental design (e.g., the presentation mode of materials, Martin & Altarriba, 2017) or the insensibility of behavioral measurement.

To further reveal the processing mechanisms of emotion-label and emotion-laden words, a body of studies adopted the event-related potential (ERP) technique with high time resolution to investigate the emotional word type effect on word processing. In ERP studies, consistent differences in the neurocognitive mechanisms underlying emotion-label and emotion-laden words have been documented (Gu & Chen, 2024b; Tang et al., 2024; Wang et al., 2019; Wu et al., 2021), and “the extended emotional word processing model” in terms of Chinese (Liu et al., 2023) and “three-stage model of bilingual emotion words processing” (Gu & Chen, 2024b) concerning these two types of emotional words have been put forward. For instance, in the model of Liu et al. (2023), they manipulated the attention allocated to the processing of emotional words. In the first stage, participants tended to differentiate the valence of words indexed by N170. In the second stage, valence interacted with emotional word type in the amplitudes of P2 and early posterior negativity (EPN). In the last stage, under the condition of explicit processing of targets, emotional word type effect was significant in the analysis of N400 and early late positive component (LPC) amplitudes and valence effect was significant only in the results of late LPC. Under the circumstance of implicit processing, the significant emotional word type effect was only found in the analysis of N400.

Nevertheless, findings regarding their neural correlates are not uniformly consistent across distinct processing stages. Specifically, during the early perceptual processing phase, there is no consensus in the literature as to whether the amplitude of the N170 component is greater for emotion-label (Zhang et al., 2017) or emotion-laden words (Liu & Fan, 2023). For studies examining word processing, N170 amplitude is linked to attention allocation, as supported by J. Zhao et al. (2012). In the semantic processing stage, EPN and N400 are often reported. The amplitude of EPN reflects the complexity of stimuli (Herbert et al., 2008) and the semantic processing of word (W. Zhao et al., 2018) and N400 relates to semantic access and expectation (Kutas & Federmeier, 2011) as well as cognitive processing of conflict in certain conditions (Imbir et al., 2018). In the elaborative processing stage, a significant effect in the amplitudes of LPC effect is reported significant (Liu et al., 2023) or absent (Liu & Fan, 2023). The LPC indicates high-level evaluation of stimuli, encoding of episodic memory (Schupp et al., 2006), and sustained elaborate processing of emotional information of stimuli (Citron, 2012).

The reasons for inconsistent findings regarding the processing of specific emotional words across different stages in previous research might be variations in the task design and the control of experimental materials. Additionally, individual differences may represent another critical contributing factor. It has been reported that trait anxiety influenced the processing of emotional stimuli, especially negative stimuli (Armitage & Eerola, 2025). Therefore, individuals with different levels of trait anxiety might behave differently when they process emotion-label and emotion-laden words since there are different semantic and affective access routes for these two types of emotional words. In addition, in empirical studies that focus on exploring the relationship between trait anxiety and emotional word processing, two common experimental paradigms—EST and dot-probe tasks—have been widely employed across different samples and settings, yet both approaches still have notable limitations in terms of ecological validity and result generalizability. These tasks measure “attentional bias” instead of the real processing of emotional words. Nevertheless, relative to the EST and dot-probe task, the lexical decision task (LDT) could reflect the “semantic processing depth” of emotional words, which minimizes confounding factors.

To further reveal the neural mechanisms underlying different stages of emotional word processing, the present study adopted an LDT to investigate the effects of trait anxiety and emotional word type on the processing of Chinese words. Trait anxiety is the between-subjects factor, comprising HTA, medium-trait-anxious (MTA), and LTA groups. Emotional word type is the within-subjects factor, consisting of emotion-label, emotion-laden, and neutral words. To the best of our knowledge, no study has investigated the interaction effects between trait anxiety and emotional word type on the processing of Chinese words. We hypothesized that there would be a significant and robust main effect of emotional word type during the semantic processing stage. During this stage, this emotional word type effect might be modulated by trait anxiety. Specifically, HTA individuals might differentiate emotion-laden from emotion-label and neutral words instead of LTA and MTA participants since they generally exhibit an attentional bias towards negative stimuli. In addition, there might be differences between HTA and LTA participants during the late processing stage of emotional words.

## 2. Materials and Method

### 2.1. Participants

Sixty-seven participants voluntarily took part in the present study. All of them finished the State-Trait Anxiety Inventory (Spielberger et al., 1983). This inventory consists of two scales to measure the state anxiety and trait anxiety scores of individuals. Each scale includes twenty items. In the scale of state anxiety, the items are scored on a 4-point scale: “NOT AT ALL”, “SOMEWHAT”, “MODERATELY SO”, “VERY MUCH SO” for 1, 2, 3, 4, respectively. Items 1, 2, 5, 8, 10, 11, 15, 16, 19, 20 are scored reversely. In the scale of trait anxiety, the items are scored on four-point scale: “ALMOST NEVER”, “SOMETIMES”, “OFTEN”, “ALMOST ALWAYS” for 1, 2, 3, 4, respectively. Reversed scores are computed for items 1, 3, 6, 7, 10, 13, 14, 16, 19.

According to the trait anxiety scores of participants, we divided them into high (40–49), medium (35–40), and low (25–35) trait anxiety groups. The participants were divided into three groups to capture potential non-linear differences in trait anxiety-related responses, rather than imposing a binary high-low classification. To address potential confounding effects of state anxiety, an exact matching procedure was then applied. Specifically, participants were first allocated based on their trait anxiety scores, and then small adjustments were made to ensure that the mean state anxiety scores did not significantly differ across the three groups. This procedure guaranteed comparability of state anxiety while preserving meaningful differences in trait anxiety. Finally, each group included twenty participants. Their trait anxiety scores decreased significantly in sequence (*p*s < 0.05), while there were no significant differences in state anxiety scores among the groups (*p*s > 0.05). The state anxiety and trait anxiety had adequate reliability (α = 0.887 and α = 0.809) in the present sample. In addition, the age of the selected sixty participants ranged from 18 to 30 (*M* = 21.30, *SD* = 2.38) and there was no significant difference in age across the three groups (*p*s > 0.05). The gender information, age, state anxiety scores, and trait anxiety scores are provided in Table 1.

All of them had normal or corrected-to-normal vision without psychological or neurological illness. One participant was left-handed; the others were right-handed. Participants signed the written informed consent before the experiments and received funds after the experiment. The experiment was approved by the Ethics Board of Artificial Intelligence and Human Languages Lab of Beijing Foreign Studies University.

### 2.2. Materials

The materials were selected from the self-constructed emotional word pool presented in Liu’s (2021) thesis. In this pool, emotion-label and emotion-laden words were decided by 30 participants via yes/no voting method, as Wang et al. (2019) carried out. Then the abstractness, familiarity, valence, and arousal of the words were rated with a 7-point Likert scale by a separate group of raters who did not participate in the formal experiment. The number of valid raters for each attribute of all words was 30. The frequency of words was from Cai and Brysbaert (2010). The current study selected 60 emotion-label, emotion-laden, and neutral words, respectively. There was no significant difference among three groups of words in terms of abstractness, familiarity, stoke number, and frequency (*p*s > 0.05). For arousal and valence, there was no significant difference between emotion-label and emotion-laden words (*p*s > 0.05). Both types of emotional words exhibited lower valence and higher arousal compared to neutral words. The attributes of the materials are displayed in Table 2. It should be noted that all emotional words in the current study were negative in valence. This decision was based on previous evidence showing that negative stimuli typically evoke stronger emotional and attentional responses, particularly in anxiety-related contexts, thereby providing greater sensitivity for detecting potential group differences (Bar-Haim et al., 2007). The current study also created 180 pseudowords which were auto-generated via rule-based formulas in Excel and were matched with real words on stroke number (*p* > 0.05) and length (two characters), being semantically meaningless. All words and pseudowords were repeated once.

### 2.3. Procedure

The experiment was conducted in an ERP Lab. Participants were instructed to finish 24 practice trials to familiarize themselves with the task. During the formal experiment, a fixation of 500 ms marked the start of a trial. Then the target word was displayed for a 1000 ms duration until a response was made. The participants were required to judge whether the target word was a real word or not as fast and accurately as possible. Press “z” if the word is a real one, otherwise press “m”. The assignment of left-hand vs. right-hand key presses was counterbalanced across participants. The response within 1500 ms would be recorded. After the response, there would be a random blank with the duration of 800–1000 ms. The whole experiment was divided into 10 blocks and the words in each block were presented randomly. Each block included 74 trials, with the first 2 trials serving as fillers to stabilize electroencephalogram (EEG) signals at the beginning of the block. Participants could take customized rest between blocks.

### 2.4. EEG Recording and Processing

The electroencephalogram (EEG) data were collected via 64-channel Ag-AgCl electrode system using an elastic cap (NeuroScan Inc., Herndon, VA, USA). The horizontal electrooculogram (EOG) was registered with two active electrodes placed to both sides of the external canthi to record eye movements. The vertical EOGs were collected with two active electrodes placed above and below the right eye to record eye blinks. The online reference was the tip of nose. The electrode impedance was kept beneath 5 kΩ. Neuroscan SynAmps amplifier was adopted to record the continuing electrophysiological data with a sample rate of 1000 Hz.

EEGLAB (Delorme & Makeig, 2004) and the EEGLAB plug-in ERPLAB (Lopez-Calderon & Luck, 2014) were adopted to analyze the data. First, the useless electrodes (M1, M2, CB1, CB2, HEO, VEO, and TRIGGER) were removed. Then, a high-pass filter at 0.1 Hz (second-order, −12 dB/octave) was applied to eliminate slow drifts, followed by a 30 Hz low-pass filter to suppress high-frequency noise. Next, a basic event list was then created and a binned event list was generated according to the specified bin file and integrated into the EEG data. Bad channels were interpolated and the data were re-referenced using an average reference. Epochs were segmented spanning from 200 ms before the onset of the display of the target word to 800 ms after this onset, with pre-event baseline correction applied. Subsequently, independent component analysis (ICA) was performed to remove artifacts evoked by eye blinks or eye movements. Brain waves outside ±100 μV were cast out. The EEG data were averaged only for trials with correct responses for subsequent analyses. There were no significant differences in average numbers among the trials for emotion-label, emotion-laden, and neutral words across HTA, MTA, and LTA groups (*p*s > 0.05).

Based on existing research and blind visual inspection of the grand averages (with inspectors blinded to all experimental conditions), the extracted ERP data were analyzed for the following time windows: 100–140 ms (P1), 170–220 ms (N170), 260–330 ms (EPN), 250–410 ms (N400) and 410–800 ms (LPC). The time window and electrode selection are presented in Table 3. For the analysis of P1, N170, and EPN, the hemisphere effect was included according to previous studies (Kissler et al., 2009; Liu et al., 2023; Zhang et al., 2017).

## 3. Results

For behavioral data, trials with incorrect responses (2.30%) and reaction times (RTs) outside the range of within-participants M ± 3 SD (1.45%) were excluded from further analysis. Repeated measures analyses of variance (ANOVAs) were performed, with group (HTA, MTA, and LTA) as the between-subjects factor and emotional word type (emotion-label, emotion-laden, and neutral words) as the within-subjects factor. For the N400 and LPC components, ANOVAs were conducted with groups as the between-subjects factor and emotional word type as the within-subjects factor. For the P1, N170, and EPN components, hemisphere was added as an additional within-subjects factor. Since the hemispheric effect was not the focus of the present research, only significant interactions between the hemispheric effect and other effects were examined, and the independent mechanisms of the hemispheric effect itself were not analyzed. A significance level of *p* < 0.05 was adopted, with the Greenhouse–Geisser epsilon correction applied when necessary. For pairwise comparisons, alpha levels were adjusted using the Bonferroni method.

### 3.1. Behavioral Data

For the analysis of accuracy (ACC), the main effect of emotional word type was significant, *F* (2, 114) = 4.083, *p* = 0.025, *η*^2^_p_ = 0.067. Specifically, the ACC of emotion-laden words (0.973) was significantly lower than that of emotion-label words (0.978, *p* = 0.05) and neutral words (0.980, *p* = 0.027) and there was no significant difference between the ACC of the emotion-label words and neutral words. For the analysis of RT, emotional word type effect was significant, *F* (2, 114) = 17.940, *p* < 0.001, *η*^2^_p_ = 0.239. The RT of emotion-laden words (555.472 ms) was significantly longer than that of emotion-label words (547.331 ms) and neutral words (549.859 ms, *p*s < 0.001), and there was no significant difference between the RT of emotion-label words and neutral words.

### 3.2. ERP Data

P1

**Figure 1:**
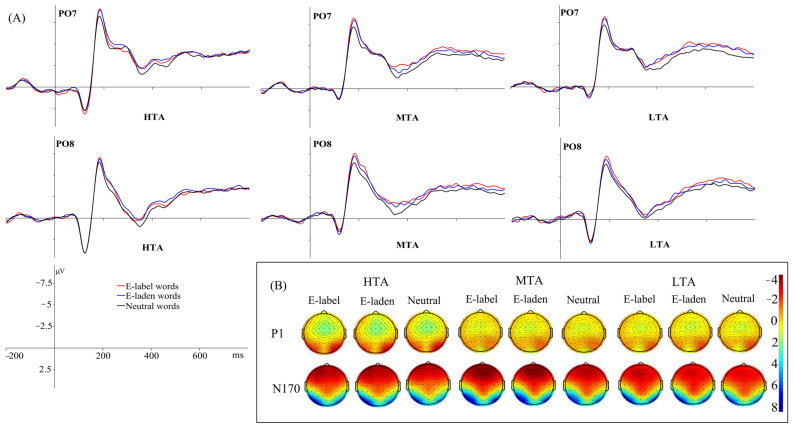
Mean grand-average ERPs (**A**) at representative electrodes (PO7 and PO8) and the topography of cortical responses (**B**) to the three word types across the three groups for the P1 and N170 components.

The interaction effect between emotional word type and hemisphere was significant, *F* (2, 114) = 5.636, *p* = 0.006, *η*^2^_p_ = 0.090. Pairwise comparison analysis revealed that across all word types, words in the right hemisphere elicited larger P1 amplitudes than those in the left hemisphere (*p*s < 0.05); in both hemispheres, there was no significant difference between emotion-label, emotion-laden, and neutral words. The overall mean P1 amplitudes and their corresponding topographic distributions of representative electrodes are illustrated in Figure 1.

N170

The main effect of emotional word type was significant, *F* (2, 114) = 49.085, *p* < 0.001, *η*^2^_p_ = 0.463. Further analysis found that both emotion-label (−5.640 μV) and emotion-laden (−5.498 μV) words elicited an enhanced N170 compared with neutral words (−4.973 μV, *p*s < 0.001), and there was no significant difference between the N170 amplitudes elicited by these two types of emotional words. The grand-average N170 amplitudes and topographic maps of representative electrodes are shown in Figure 1.

EPN

Emotional word type effect was significant, *F* (2, 114) = 4.838, *p* = 0.011, *η*^2^_p_ = 0.078. Pairwise comparison analysis found that emotion-laden words (−2.039 μV) elicited larger EPN amplitudes than neutral words (−1.787 μV, *p* = 0.006), with no significant differences between emotion-label (−1.867 μV) and emotion-laden words, or between emotion-label and neutral words (*p*s > 0.05).

The interaction effect between emotional word type and group was significant, *F* (2, 114) = 2.502, *p* = 0.049, *η*^2^_p_ = 0.081. Further analysis revealed that for the HTA group, emotion-laden words (−2.155 μV) elicited an enhanced EPN relative to emotion-label (−1.622 μV) and neutral (−1.753 μV) words (*p*s < 0.05), and emotion-label and neutral words elicited comparable EPN amplitudes (*p* > 0.1). For the MTA and LTA groups, there was no significant difference among different types of words (*p*s > 0.05). For all word types, EPN amplitudes elicited in the three groups were not found to differ significantly (*p*s > 0.05).

**Figure 2:**
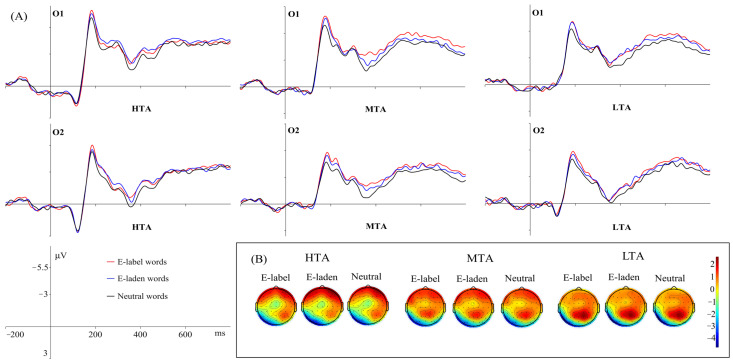
Mean grand-average ERPs (**A**) at representative electrodes (O1 and O2) and the topography of cortical responses (**B**) to the three word types across the three groups for the EPN components.

The interaction effect between emotional word type and hemisphere was significant, *F* (2, 114) = 6.493, *p* = 0.002, *η*^2^_p_ = 0.102. Further analysis found that in the right hemisphere, emotion-laden words (−1.103 μV) evoked an enhanced EPN compared with neutral words (−0.743 μV, *p* < 0.001), and there were no significant differences between emotion-label (−0.941 μV) and emotion-laden words, or between emotion-label and neutral words; for the three types of words, words in the left hemisphere produced larger EPN than those in the right hemisphere (*p*s < 0.001). For all word types, the EPN amplitudes elicited in the left hemisphere were significantly larger than those elicited in the right hemisphere (*p*s < 0.001). The EPN grand-average amplitudes and associated scalp topographies of representative electrodes can be seen in Figure 2.

N400

**Figure 3:**
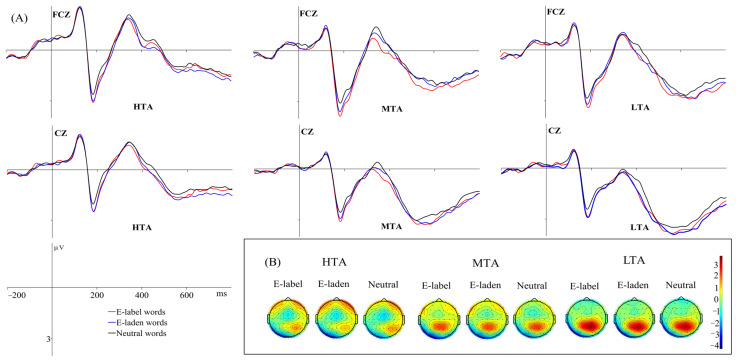
Mean grand-average ERPs (**A**) at representative electrodes (FCZ and CZ) and the topography of cortical responses (**B**) to the three word types across the three groups for the N400 components.

The main effect of emotional word type was significant, *F* (2, 114) = 8.062, *p* < 0.001, *η*^2^_p_ = 0.124. Pairwise comparisons revealed that emotion-label words (0.232 μV) produced a weaker N400 than neutral words (0.003 μV, *p* < 0.001), and there was no significant difference between emotion-label and emotion-laden (0.123 μV) words, or between emotion-laden and neutral words. Figure 3 shows the mean N400 amplitudes across participants and the corresponding topographical maps of representative electrodes.

LPC

The main effect of emotional word type was significant, *F* (2, 114) = 5.314, *p* = 0.007, *η*^2^_p_ = 0.085. Pairwise comparisons indicated that emotion-laden words (1.447 μV) evoked larger LPC amplitudes than neutral words (1.275 μV, *p* = 0.004), and there was no significant difference between emotion-label (1.316 μV) and emotion-laden words, or between emotion-label and neutral words (*p*s > 0.05).

**Figure 4:**
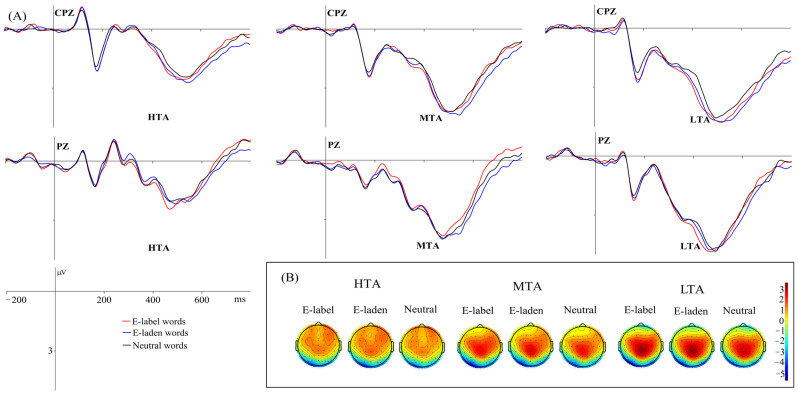
Mean grand-average ERPs (**A**) at representative electrodes (CPZ and PZ) and the topography of cortical responses (**B**) to the three word types across the three groups for the LPC components.

The main effect of group was significant, *F* (2, 57) = 5.363, *p* = 0.007, *η*^2^_p_ = 0.158. Pairwise comparison analysis showed that words processed by the LTA group (1.975 μV) produced larger LPCs than those processed by the HTA group (0.739 μV, *p* = 0.005), and there was no significant difference between HTA and MTA (1.324 μV) groups, or between LTA and MTA groups (*p*s > 0.05). Figure 4 depicts the grand-average LPC amplitudes along with their scalp topography of representative electrodes.

## 4. Discussion

Both behavioral and electrophysiological data showed a significant and robust main effect of emotional word type which supported our first hypothesis. Behaviorally, emotion-laden words were associated with lower ACC and longer RTs than emotion-label and neutral words. This finding was consistent with those of previous studies (Gu & Chen, 2024a; Kazanas & Altarriba, 2015).

The ERP results showed that, in the perceptual processing stage, emotion-label and emotion-laden words elicited enhanced N170 amplitudes relative to neutral ones, and there was no significant difference between the N170 amplitudes of emotion-label and emotion-laden words. This result indicated that compared to neutral words, emotional words could attract the early attention of individuals. This finding was similar to that of Liu et al. (2023), which revealed that only the valence effect was significant during this stage. However, this result was inconsistent with the findings of Zhang et al. (2017), who reported that emotion-label words elicited larger N170 amplitudes than emotion-laden words in the right hemisphere, as well as with those of Liu and Fan (2023), who found that emotion-laden words evoked stronger N170 amplitudes than emotion-label ones. The effect direction was inconsistent in the N170 component, which might be due to a comprehensive influence of many factors, including the selection of task and the control of concreteness, and valence attributes of materials.

In the semantic processing stage, the EPN and N400 components were observed. In the right hemisphere, emotion-laden words elicited greater EPN amplitudes than neutral words, and there were no significant differences in EPN amplitudes between emotion-laden and emotion-label words, nor between emotion-label and neutral words. This finding was different from prior studies. In both ECT and EST, Liu et al. (2023) found emotion-laden words elicited boosted EPN amplitudes relative to emotion-label words. A similar result was reported in the LDT of Tang et al. (2024). In general, EPN reflects the complexity of stimuli (Herbert et al., 2008). Emotion-laden words carry both affective and semantic meanings, and they are more complex than emotion-label and neutral words. In our study, both emotion-label and emotion-laden words were negative ones, which may align with previously reported negative bias (Mogg et al., 2000). Individuals had difficulty in disengaging themselves away from negative stimuli (Armstrong & Olatunji, 2012). Therefore, there was no significant difference between the EPN amplitudes of these two types of emotional words.

For the N400 component, emotion-label words produced a weaker N400 than neutral words, and there were no significant differences between emotion-label and emotion-laden words as well as between emotion-laden and neutral ones. This result was in support of the finding of Wang et al. (2019) which reported that negative emotion-label, negative emotion-laden, positive emotion-label and positive emotion-laden words elicited weaker N400 amplitudes than neutral words in an LDT, and there was no noteworthy difference between the N400 amplitudes of emotion-label and emotion-laden words. The findings indicated that emotional words were associated with more facilitated semantic integration than neutral ones (Liu et al., 2023). In the ECT, Liu et al. (2023) found similar N400 amplitudes for emotion-label and emotion-laden words; in the EST, they reported a significantly stronger N400 was evoked by emotion-label words than emotion-laden words, demonstrating a facilitated semantic integration for emotion-label words. A similar semantic integration facilitation effect was also reported in a flanker task (Wu & Zhang, 2019).

In the elaborative processing stage of Chinese words, emotion-laden words elicited larger LPC amplitudes than neutral ones, and there were no significant differences between the LPC amplitudes of emotion-laden and emotion-label words, or between emotion-label and neutral words. The finding demonstrated a continuous detailed processing of the affective information in emotion-laden words since they have both affective and semantic meanings. Existing studies also reported that the LPC amplitudes elicited by emotion-label and emotion-laden words showed no significant difference (Liu & Fan, 2023; Liu et al., 2023).

Our second hypothesis was evidenced by the finding that the effect of emotional word type was modulated by group. For HTA individuals, emotion-laden words evoked a larger EPN amplitude than emotion-label and neutral words, and the EPN amplitudes between emotion-label and neutral words showed no significant difference. MTA and LTA individuals showed no such differences in the EPN amplitude. That emotion-laden words produced enhanced EPN amplitudes compared with emotion-label ones was consistent with previous studies (Liu & Fan, 2023; Tang et al., 2024), supporting the mediated emotion concept account (Altarriba & Basnight-Brown, 2010), density hypothesis (Unkelbach et al., 2008; Unkelbach et al., 2010) as well as the account of embodiment of emotion (Tang et al., 2023; Vigliocco et al., 2009). The mediated emotion concept account holds the point of view that compared to emotion-label words, emotion-laden words access the emotion concept through the mediation of a certain “event”. For example, the emotion concept of emotion-label word “sad” could be directly accessed, while the emotion concept of emotion-laden word “coffin” would be accessed through the mediation of the concept of “coffin”. There would be more steps to access the affective concept of emotion-laden words. The density hypothesis was first used to explain the processing difference of positive and negative words, and Zhang et al. (2019) adopted it to explain the differences between emotion-label and emotion-laden words. According to them, the emotion concepts of emotion-label words demonstrated a much higher density than those of emotion-laden words. For instance, the emotion concept(s) of “happy” was “happy”, while the emotion concept(s) of “reward” could be “happy” “proud” “satisfying” and so on. The high semantic density grasped more attention from individuals; therefore, emotion-laden words could elicit a stronger EPN amplitude than emotion-label words. Similarly to emotion-label words, the affective meaning and semantic meaning of neutral words were the same as those of emotion-label words, leading to no significant differences between the EPN amplitudes of emotion-label words and neutral words. Additionally, emotion-laden words have been evidenced to be affectively disembodied (Tang et al., 2023). The processing of emotion-label words was more embodied than emotion-laden ones because they were encountered more frequently in daily life (Tang et al., 2024).

The emotional word type effect was observed exclusively in HTA individuals, whereas no such effect was detected in MTA or LTA individuals. This might be because the emotional words in the present study were all negative ones. HTA participants paid more attention to negative stimuli (Miller & Patrick, 2000) and showed a stronger attentional bias towards negative information (Edwards et al., 2010). In addition, according to attentional control theory (Eysenck et al., 2007), anxiety could undermine the efficiency of the goal-directed attentional system and endow the stimulus-driven attentional system with greater influence on processing. In other words, relative to MTA and LTA participants, HTA participants were more susceptible to the stimulus-driven system due to their higher level of anxiety. Compared to emotion-label and neutral words, emotion-laden words carry both affective and semantic information, making them inherently more salient. In contrast, the anxiety level of MTA and LTA participants was lower and the goal-directed attentional system was not impaired. They could complete the LDT under the full control of the goal-directed attentional system with less interference from different types of stimuli. This may explain why the modulatory effect of emotional word type on the EPN amplitudes was only manifested in HTA individuals rather than MTA and LTA individuals in the current study.

The last hypothesis was supported by a significant group effect in the amplitudes of LPC. Surprisingly, the LPC amplitudes of LTA individuals were noteworthily larger than those of HTA participants, and there was no significant difference between the LPC amplitudes of LTA and MTA individuals as well as between MTA and HTA individuals. This seemingly paradoxical result may be related to context-dependent impairments in cognitive control among individuals with HTA. Impoverished recruitment of prefrontal attentional control mechanisms in HTA participants was reported, even when threatening stimuli were absent (Bishop, 2009). The recruitment of prefrontal mechanisms was important for active management of attention (Bishop, 2009). The negative stimuli in the current study might interfere with the recruitment of prefrontal attentional control mechanisms of HTA individuals in the late processing stage. That is to say, HTA individuals demonstrated the “vigilance-avoidance” pattern for the processing of negative stimuli. At first, they automatically allocated attention to the negative targets, and then showed an avoidance of them (Koster et al., 2005). This explanation was evidenced by the result of EPN and LPC. At an earlier semantic processing stage, HTA participants had full access to the affective and semantic meanings of the stimuli as indexed by EPN, and this evoked automatic vigilance towards the target words. After this, HTA individuals activated the avoidance system to reduce the anxiety evoked by the stimuli. On the other hand, LTA participants could have an appropriate recruitment of prefrontal attentional control mechanisms to conduct elaborative analysis of the targets at the late processing stage. Hence, HTA individuals exhibited significantly smaller LPC amplitudes than LTA individuals.

The above findings evidenced the classification of three stages of emotional word processing models and the N170 effect replicated the first processing stage of the extended emotional word processing model of Liu et al. (2023) to a certain degree. However, as the model proposed, the level of attention allocation would modulate the processing of emotional words. The attentional demands of the LDT differed from those of the ECT and EST. Consequently, ERP components elicited by emotional words were likely to vary across different processing stages. In addition, the present study also found that individual differences, particularly trait anxiety of participants, played an important role in emotional word processing. HTA individuals had the tendency to automatically attend to negative stimuli at an earlier processing stage, and then they avoided processing them further to reduce their anxiety. That is to say, trait anxiety of individuals modulated emotional word processing in different stages, and the construction of emotional word processing models should take individual differences into consideration.

## 5. Conclusions

The present study adopted an LDT to examine the effects of trait anxiety and emotional word type on the processing of Chinese words, with state anxiety controlled. Behaviorally, emotion-laden words were associated with longer RTs and lower ACC than emotion-label and neutral words. Electrophysiologically, both emotion-label and emotion-laden words elicited a larger N170 amplitude than neutral words. Compared to neutral words, emotion-laden words evoked larger EPN amplitudes in the right hemisphere and enhanced LPC amplitudes. Emotion-label words produced stronger N400 amplitudes than neutral ones. The EPN amplitudes were modulated by the interaction between the level of trait anxiety and emotional word type. Emotion-laden words evoked larger EPN amplitudes than emotion-label and neutral words in HTA individuals, which aligns with the mediated emotion concept account, density hypothesis and embodiment emotion account. In the late elaborated processing stage, LTA participants were associated with larger LPC amplitudes than HTA individuals, which was consistent with the “vigilance-avoidance” pattern.

The present study provided implications for the construction of emotional word processing models by indicating the interaction between emotional word type and trait anxiety. Practically, specific training on attention or emotion regulation might be developed to relieve the anxiety of HTA individuals when they encountered negative stimuli. In addition, in language teaching, teachers should guide students to pay attention to the dissociation between emotion-label and emotion-laden words and create embodied contexts for learning emotional words, particularly emotion-laden words.

However, there were some limitations in the current study. First, the major limitation of the current study was that only negative and neutral words were adopted. Even though the design choice increased experimental sensitivity and was theoretically justified, it inevitably limits how broadly the results can be applied. Thus, the observed effects may reflect processing bias toward negative emotional information instead of emotional stimuli in general. Future research should include positive emotional words to investigate whether the current findings can be generalized beyond negativity-specific responses. Secondly, although significant differences in trait anxiety scores were observed among the HTA, MTA, and LTA groups, future studies could recruit more participants and further increase the score differences between groups to better investigate the modulatory effect of trait anxiety on emotional word processing. Third, this study only examined trait anxiety as an individual difference variable during the processing of emotional words. It would be more comprehensive for future research to investigate the effects of other individual difference variables such as interoception and gender on the mechanisms of emotional word processing.

## Figures and Tables

**Table 1:** The gender information, age, state anxiety scores, and trait anxiety scores of the participants.

Group	Gender	Number	Age	S-Anxiety Scores	T-Anxiety Scores
HTA	female	11	21.35	33.10	43.80
	male	9			
MTA	female	9	21.40	30.50	37.85
	male	11			
LTA	female	10	21.45	28.35	32.25
	male	10			

Note: HTA = high-trait-anxious, MTA = medium-trait-anxious, LTA = low-trait-anxious, S-Anxiety = state anxiety, T-Anxiety = trait anxiety.

**Table 2:** The descriptive data of the attributes of the materials.

Word Type	Frequency	Familiarity	Arousal	Valence	Abstractness	Stroke Number
E-label words	16.40 (25.59)	6.07 (0.24)	4.77 (0.52)	2.22 (0.42)	3.70 (0.43)	19.22 (4.87)
E-laden words	19.29 (29.45)	6.07 (0.27)	4.73 (0.54)	2.25 (0.39)	3.53 (0.66)	17.78 (4.88)
Neutral words	15.38 (13.1)	5.98 (0.3)	2.61 (0.16)	4.06 (0.28)	3.66 (0.55)	18.05 (3.63)

Note: E-label = emotion-label; E-laden = emotion-laden.

**Table 3:** The time window and electrode selection for each ERP components.

	Time Window (ms)	Electrodes	
P1	100–140	LH: PO3, PO5, PO7	RH: PO4, PO6, PO8
N170	170–220	LH: P5, P7, PO5, PO7	RH: P6, P8, PO6, PO8
EPN	260–330	LH: PO3, PO5, O1	RH: PO4, PO6, O2
N400	300–400	FC1, FCZ, FC2, C1, CZ, C2	
LPC	410–800	CP1, CP2, CPZ, CP3, CP4, P1, P2, PZ, P3, P4	

Note: LH = Left Hemisphere, RH = Right Hemisphere.

## Data Availability

The data presented in this study are available on request from the corresponding author.

## References

[B1-behavsci-16-00096] Altarriba J., Basnight-Brown D. M. (2010). The representation of emotion vs. emotion-laden words in English and Spanish in the affective Simon task. International Journal of Bilingualism.

[B2-behavsci-16-00096] Armitage J., Eerola T. (2025). Auditory affective priming: The role of trait anxiety and stimulus type. Psychology of Music.

[B3-behavsci-16-00096] Armstrong T., Olatunji B. O. (2012). Eye tracking of attention in the affective disorders: A meta-analytic review and synthesis. Clinical Psychology Review.

[B4-behavsci-16-00096] Bar-Haim Y., Lamy D., Pergamin L., Bakermans-Kranenburg M. J., van I. M. H. (2007). Threat-related attentional bias in anxious and nonanxious individuals: A meta-analytic study. Psychological Bulletin.

[B5-behavsci-16-00096] Bishop S. (2009). Trait anxiety and impoverished prefrontal control of attention. Nature Neuroscience.

[B6-behavsci-16-00096] Cai Q., Brysbaert M. (2010). SUBTLEX-CH: Chinese word and character frequencies based on film subtitles. PLoS ONE.

[B7-behavsci-16-00096] Citron F. M. M. (2012). Neural correlates of written emotion word processing: A review of recent electrophysiological and hemmodynamic neuroimaging studies. Brain and Language.

[B8-behavsci-16-00096] Delorme A., Makeig S. (2004). EEGLAB: An open source toolbox for analysis of single-trial EEG dynamics including independent component analysis. Journal of Neuroscience Methods.

[B9-behavsci-16-00096] Edwards M. S., Burt J. S., Lipp O. V. (2006). Selective processing of masked and unmasked verbal threat material in anxiety: Influence of an immediate acute stressor. Cognition and Emotion.

[B10-behavsci-16-00096] Edwards M. S., Burt J. S., Lipp O. V. (2010). Selective attention for masked and unmasked threatening words in anxiety: Effects of trait anxiety, state anxiety and awareness. Behaviour Research and Therapy.

[B11-behavsci-16-00096] Eysenck M. W., Derakshan N., Santos R., Calvo M. G. (2007). Anxiety and cognitive performance: Attentional control theory. Emotion.

[B12-behavsci-16-00096] Gu X., Chen S. (2024a). Emotion in language: Emotion word type and valence interactively predicted Chinese emotional word processing in emotion categorization task. International Journal of Applied Linguistics.

[B13-behavsci-16-00096] Gu X., Chen S. (2024b). Two languages, two emotional minds in one brain: Processing emotion-label and emotion-laden words by Chinese-English bilinguals. International Journal of Bilingual Education and Bilingualism.

[B14-behavsci-16-00096] Herbert C., Junghofer M., Kissler J. (2008). Event related potentials to emotional adjectives during reading. Psychophysiology.

[B15-behavsci-16-00096] Imbir K. K., Jurkiewicz G., Duda-Golawska J., Pastwa M., Zygierewicz J. (2018). The N400/FN400 and lateralized readiness potential neural correlates of valence and origin of words’ affective connotations in ambiguous task processing. Frontiers in Psychology.

[B16-behavsci-16-00096] Kazanas S., Altarriba J. (2015). The automatic activation of emotion and emotion-laden words: Evidence from a masked and unmasked priming paradigm. The American Journal of Psychology.

[B17-behavsci-16-00096] Kissler J., Herbert C., Winkler I., Junghofer M. (2009). Emotion and attention in visual word processing: An ERP study. Biological Psychology.

[B18-behavsci-16-00096] Knickerbocker H., Johnson R. L., Altarriba J. (2015). Emotion effects during reading: Influence of an emotion target word on eye movements and processing. Cognition and Emotion.

[B19-behavsci-16-00096] Koster E. H. W., Verschuere B., Crombez G., Van Damme S. (2005). Time-course of attention for threatening pictures in high and low trait anxiety. Behaviour Research and Therapy.

[B20-behavsci-16-00096] Kutas M., Federmeier K. D. (2011). Thirty years and counting: Finding meaning in the N400 component of the event-related brain potential (ERP). Annual Review of Psychology.

[B21-behavsci-16-00096] Liu J. (2021). A study of the attention-oriented neural mechanisms of Chinese emotion-label and emotion-laden words. Ph.D. thesis.

[B22-behavsci-16-00096] Liu J., Fan L. (2023). An ERP study on Chinese emotion-label and emotion-laden word processing. Journal of Foreign Languages.

[B23-behavsci-16-00096] Liu J., Fan L., Tian L., Li C., Feng W. (2023). The neural mechanisms of explicit and implicit processing of Chinese emotion-label and emotion-laden words: Evidence from emotional categorization and emotional Stroop tasks. Language, Cognition and Neuroscience.

[B24-behavsci-16-00096] Lopez-Calderon J., Luck S. J. (2014). ERPLAB: An open-source toolbox for the analysis of event-related potentials. Frontiers in Human Neuroscience.

[B25-behavsci-16-00096] Macleod C., Hagan R. (1992). Individual differences in the selective processing of threatening information, and emotional responses to a stressful life event. Behaviour Research and Therapy.

[B26-behavsci-16-00096] MacLeod C., Rutherford E. M. (1992). Anxiety and the selective processing of emotional information: Mediating roles of awareness, trait and state variables, and personal relevance of stimulus materials. Behaviour Research and Therapy.

[B27-behavsci-16-00096] Martin J. M., Altarriba J. (2017). Effects of valence on hemispheric specialization for emotion word processing. Language and Speech.

[B28-behavsci-16-00096] Miller M. W., Patrick C. J. (2000). Trait differences in affective and attentional responding to threat revealed by emotional stroop interference and startle reflex modulation. Behavior Therapy.

[B29-behavsci-16-00096] Mogg K., McNamara J., Powys M., Rawlinson H., Seiffer A., Bradley B. P. (2000). Selective attention to threat: A test of two cognitive models of anxiety. Cognition & Emotion.

[B30-behavsci-16-00096] Pavlenko A. (2008). Emotion and emotion-laden words in the bilingual lexicon. Bilingualism: Language and Cognition.

[B31-behavsci-16-00096] Rutherford E. M., MacLeod C., Campbell L. W. (2004). Negative selectivity effects and emotional selectivity effects in anxiety: Differential attentional correlates of state and trait variables. Cognition and Emotion.

[B32-behavsci-16-00096] Schupp H. T., Flaisch T., Stockburger J., Junghofer M. (2006). Emotion and attention: Event-related brain potential studies. Progress in Brain research.

[B33-behavsci-16-00096] Spielberger C. D., Gorsuch R. L., Lushene R., Vagg P. R., Jacobs G. A. (1983). Manual for the state-trait-anxiety inventory: STAI (form Y).

[B34-behavsci-16-00096] Tang D., Fu Y., Wang H., Liu B., Zang A., Kärkkäinen T. (2023). The embodiment of emotion-label words and emotion-laden words: Evidence from late Chinese-English bilinguals. Frontiers in Psychology.

[B35-behavsci-16-00096] Tang D., Li X., Fu Y., Wang H., Li X., Parviainen T., Kärkkäinen T. (2024). Neural correlates of emotion-label vs. emotion-laden word processing in late bilinguals: Evidence from an ERP study. Cognition and Emotion.

[B36-behavsci-16-00096] Unkelbach C., Fiedler K., Bayer M., Stegmuller M., Danner D. (2008). Why positive information is processed faster: The density hypothesis. Journal of Personality and Social Psychology.

[B37-behavsci-16-00096] Unkelbach C., von Hippel W., Forgas J. P., Robinson M. D., Shakarchi R. J., Hawkins C. (2010). Good things come easy: Subjective exposure frequency and the faster processing of positive information. Social Cognition.

[B38-behavsci-16-00096] Vigliocco G., Meteyard L., Andrews M., Kousta S. (2009). Toward a theory of semantic representation. Language and Cognition.

[B39-behavsci-16-00096] Vinson D., Ponari M., Vigliocco G. (2014). How does emotional content affect lexical processing?. Cognition and Emotion.

[B40-behavsci-16-00096] Wang X., Shangguan C., Lu J. (2019). Time course of emotion effects during emotion-label and emotion-laden word processing. Neuroscience Letters.

[B41-behavsci-16-00096] Wu C., Zhang J. (2019). Conflict processing is modulated by positive emotion word type in second language: An ERP study. Journal of Psycholinguistic Research.

[B42-behavsci-16-00096] Wu C., Zhang J., Yuan Z. (2021). Exploring affective priming effect of emotion-label words and emotion-laden words: An event-related potential study. Brain Sciences.

[B43-behavsci-16-00096] Zhang J., Wu C., Meng Y., Yuan Z. (2017). Different neural correlates of emotion-label words and emotion-laden words: An ERP study. Frontiers in Human Neuroscience.

[B44-behavsci-16-00096] Zhang J., Wu C., Yuan Z., Meng Y. (2019). Differentiating emotion-label words and emotion-laden words in emotion conflict: An ERP study. Experimental Brain Research.

[B45-behavsci-16-00096] Zhao J., Li S., Lin S. E., Cao X. H., He S., Weng X. C. (2012). Selectivity of N170 in the left hemisphere as an electrophysiological marker for expertise in reading Chinese. Neuroscience Bulletin.

[B46-behavsci-16-00096] Zhao W., Chen L., Zhou C., Luo W. (2018). Neural correlates of emotion processing in word detection task. Frontiers in Psychology.

[B47-behavsci-16-00096] Zhao Y., Jia X., Pan S., Ji H., Wang Y. (2023). Content specificity of attentional bias to COVID-19 threat-related information in trait anxiety. Frontiers in Psychiatry.

